# Precipitation Increases the Occurrence of Sporadic Legionnaires’ Disease in Taiwan

**DOI:** 10.1371/journal.pone.0114337

**Published:** 2014-12-04

**Authors:** Nai-Tzu Chen, Mu-Jean Chen, Chao-Yu Guo, Kow-Tong Chen, Huey-Jen Su

**Affiliations:** 1 Department of Environmental and Occupational Health, College of Medicine, National Cheng Kung University, Tainan, Taiwan; 2 National Environmental Health Research Center, National Health Research Institutes, Miaoli, Taiwan; 3 Institution of Public Health & Department of Public Health, College of Medicine, National Yang-Ming University, Taipei, Taiwan; 4 Department of Public Health, College of Medicine, National Cheng Kung University, Tainan, Taiwan; Queen Mary Hospital, the University of Hong Kong, Hong Kong

## Abstract

Legionnaires’ disease (LD) is an acute form of pneumonia, and changing weather is considered a plausible risk factor. Yet, the relationship between weather and LD has rarely been investigated, especially using long-term daily data. In this study, daily data was used to evaluate the impacts of precipitation, temperature, and relative humidity on LD occurrence in Taiwan from 1995–2011. A time-stratified 2:1 matched-period case-crossover design was used to compare each case with self-controlled data using a conditional logistic regression analysis, and odds ratios (ORs) for LD occurrence was estimated. The city, gender and age were defined as a stratum for each matched set to modify the effects. For lag day- 0 to 15, the precipitation at lag day-11 significantly affected LD occurrence (*p*<0.05), and a 2.5% (95% CIs = 0.3–4.7%) increased risk of LD occurrence was associated with every 5-mm increase in precipitation. In addition, stratified analyses further showed that positive associations of precipitation with LD incidence were only significant in male and elderly groups and during the warm season ORs = 1.023–1.029). However, such an effect was not completely linear. Only precipitations at 21–40 (OR = 1.643 (95% CIs = 1.074–2.513)) and 61–80 mm (OR = 2.572 (1.106–5.978)) significantly increased the risk of LD occurrence. Moreover, a negative correlation between mean temperature at an 11-day lag and LD occurrence was also found (OR = 0.975 (0.953–0.996)). No significant association between relative humidity and LD occurrence was identified (*p*>0.05). In conclusion, in warm, humid regions, an increase of daily precipitation is likely to be a critical weather factor triggering LD occurrence where the risk is found particularly significant at an 11-day lag. Additionally, precipitation at 21–40 and 61–80 mm might make LD occurrence more likely.

## Introduction

Legionnaires’ disease (LD) is a severe form of pneumonia with an overall fatality rate of 6.5–26% [1]–[4], and the mortality rate for nosocomial infections may approach 31.7–70% [5]–[7]. LD and Pontiac fever (PF) are two clinical syndromes of legionellosis caused by legionellae, while PF is a mild flu-like illness that usually clears up within 2–3 days without medical treatment [8]. People are infected through inhalation of legionellae aerosolized from man-made and natural aquatic systems as well as soil [9]–[11]. However, the growth and transmission of legionellae are likely to be affected by meteorological factors (*e.g.* temperature), and these may thereby further influences LD incidence.

Fisman et al. found a positive correlation between the occurrence of LD cases and monthly average temperature [12]. Moreover, the highest LD incidence was observed when the weather was warm and wet during the summer season [13]. Relative humidity (RH) and precipitation have also been considered possible risk factors for LD/legionellosis occurrence. Karagiannis et al. reported a 1% increase in mean weekly RH to be associated with a rising LD incidence of 5.1% [13], and an increase in monthly precipitation and mean weekly rainfall intensity have both been found to be linked to increase legionellosis/LD incidence [13]–[14]. Moreover, Fisman et al. defined an acute association with precipitation 6–10 days before legionellosis occurrence [12]. However, the study of Ng et al., [15] reported pressure, humidity, and river and creek levels as the most significant risk factors related to incidence of legionellosis rather than precipitation.

Although previously studies have suggested relationships between LD/legionellosis and meteorological factors [12]–[18], most used short-term data (about 4–9 years) [12]–[13], [16]–[17], thus lacking representation of any long-term trend. Moreover, all such studies were performed in Western countries [12]–[18], which may not apply to regions in Asia because of the differing meteorological conditions and lifestyles between Western and Asian countries. Taiwan, located in East Asia, consists of both plain and mountainous area covering a significant portion of the landscape, offering highly varied meteorological conditions as a result of straddling both the tropical and subtropical zones. Consequently, this study was aimed toward evaluating the impacts of meteorology on the occurrence of reported sporadic LD in Taiwan using long-term daily data, and an effort was made to identify the precipitation level that increases LD risk the most.

## Methods

### Case Definition of Legionnaires’ disease

We applied the infectious disease registry database provided by the Taiwan Center of Disease Control (Taiwan CDC). Since the infectious disease registry database is a secondary database without detailed personal information (*e.g.* ID number and address), all data were analyzed anonymously. The computerized database with the recorded daily registry of LD occurrence included the age, gender, township of residence, and the time of disease onset for each case, which was retrieved from the Taiwan CDC for the period from 1995–2011 using a signed confidentiality agreement. All confirmed LD cases had to meet the clinical criterion and at least one laboratory criterion (Taiwan CDC), resulting in 1,267 diagnosed cases confirmed by the reference laboratory at the Research and Diagnostic Center of the Taiwan CDC. **Clinical criteria:** symptoms include malaise, chills, myalgia, headache, fever, nonproductive cough, nausea, abdominal pain, but are led by pneumonia as the major symptom. Laboratory diagnosis: (1) isolation (culture) of legionellae species from clinical specimens (respiratory secretions, sputum, or pleural fluid); (2) detection of legionella antigen in urine samples; (3) indirect immunofluorescence antibody: diagnosis is made by the observation of a significant 4-fold increase in antibody titer to ≧128 against legionellae between sera taken in the acute phase and during convalescence four to 12 weeks later. Only “indigenous” cases for each disease, after verification of travel history, disease onset, and pathogen subtype classification by the National Virus Diagnosis Laboratory in Taiwan, were included. Moreover, no outbreak was identified by the Taiwan CDC during the period from 1995–2011, indicating that all of the reported LD cases included in our study were sporadic cases.

### Meteorological data

Hourly meteorological data from 33 fixed-site Taiwan Central Weather Bureau (Taiwan CWB) monitoring stations that had complete relative humidity, precipitation and temperature records from 1995 to 2011 were obtained.

### Statistical analysis

This study utilized a case-crossover approach to evaluate the associations between individual case occurrences of LD and weather variations. A time-stratified 2:1 matched-period case-crossover design was used. Risk periods (person-time during which the event occurred) were defined according to the date of LD symptom onset. For the purpose of comparison, there were two specific time points identified as control days; one at 7 days before, and the other at 14 days before the registry day for each case. Therefore, each analytical stratum consisted of one case day and two control days. To consider the incubation periods of LD (about 5–10 days) in the analysis, the case days that potentially corresponded to 0–15 days lag of weather exposure were tested. The estimated odds ratios (ORs) for the occurrence of cases, based on weather effects, were approximated through construction of conditional logistic regression models. The city, gender and age were defined as a stratum for each matched set. Data were also stratified by age (<50 years and ≧50 years), gender (male and female), and season (warm: Sep–Dec and Jan–Feb; cool: Mar–Aug) to further assess the impacts of individual characteristics and season on the relationships between precipitation at lag day-10 to –13 and LD occurrence. Further, the precipitation was further categorized by each 20-mm increase up to 100 mm to assess the associations between reported LD cases and precipitation levels 10–12 days prior to onset. Statistical analyses were performed with SAS version 9.3 (SAS Institute, Cary, NC).

## Results

### Legionnaires’ disease in Taiwan

**Figure 1:**
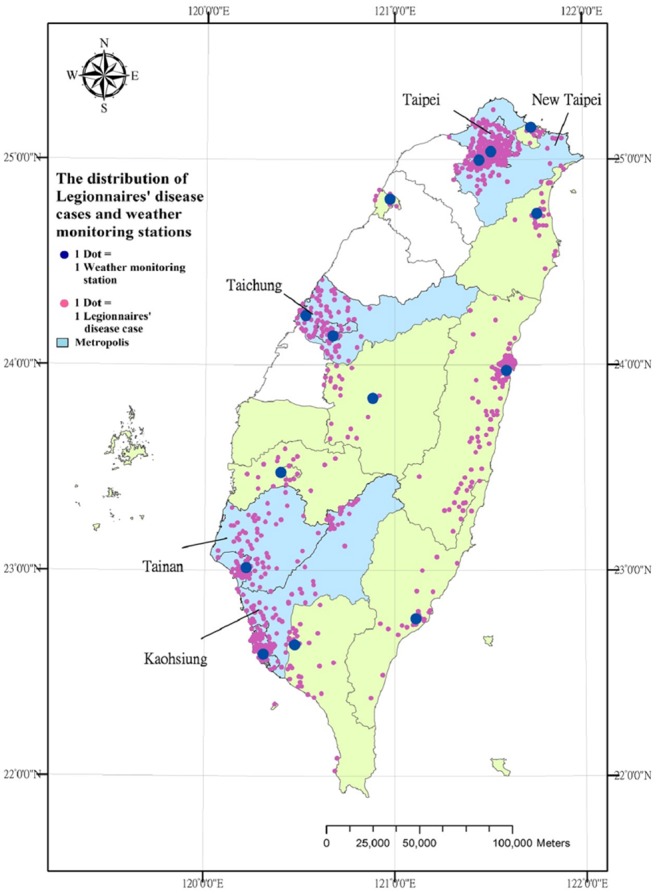
The distribution of Legionnaires’ disease cases and weather monitoring stations in Taiwan (131 cases residing in 5 cities where the weather monitoring stations were not set up).

There were 1,188 cases of LD reported between 1995 and 2011 in Taiwan. Among these 1,188 cases, males older than 65 years had the highest percentage of reported LD (38.6%), and the LD occurrence significantly differed among groups classified according to gender and age (χ^2^ = 8.23, *p*-value = 0.016) (Table 1). Except for Hualien, most cases were reported in Western Taiwan, especially in the five major cities, Taipei, New Taipei, Taichung, Tainan, and Kaohsiung, which are crowded, urbanized and have a high density of central air conditioning systems (Figure 1). By considering 131 cases residing in five cities where no weather monitoring stations were set up, only 1,057 cases were finally included in the conditional logistic regression model.

**Table 1:** Characteristics of Legionnaires’ disease cases (n = 1,188) and chi-squared distribution test in Taiwan, 1995–2011.

Gender	Age			χ^2^
	0–18	19–64	65+	(*p*-value)
**Female**	7 (0.6%)	171 (14.4%)	175 (14.7%)	8.230 (0.016)
**Male**	4 (0.3%)	372 (31.3%)	459 (38.6%)	

### Meteorological distribution

The temperature and RH ranges were 8.8–31.7°C and 41.0–99.7% respectively (Table 2). In addition, the 25^th^ percentile for the temperature was 19.8°C, and the 5^th^ percentile of RH was 63%, thus illustrating the characteristics of a warm, humid climate in Taiwan. Moreover, with precipitation ranging from 0 to 462.3 mm, and nearly half of the year exhibiting drought conditions (0 mm), a large variation in precipitation distribution was apparent.

**Table 2:** The distribution of weather factors in 14 cities, 1995–2011.

Variable	Min.	5^th^	25^th^	50^th^	75^th^	90^th^	Max.
Temperature	8.8	15.1	19.8	24.1	27.3	28.8	31.7
Relative humidity	41	63	72	77	82	87	100
Precipitation	0.0	0.0	0.0	0.1	2.3	15.2	462.3

### Associations of meteorology with reported LD cases

**Figure 2:**
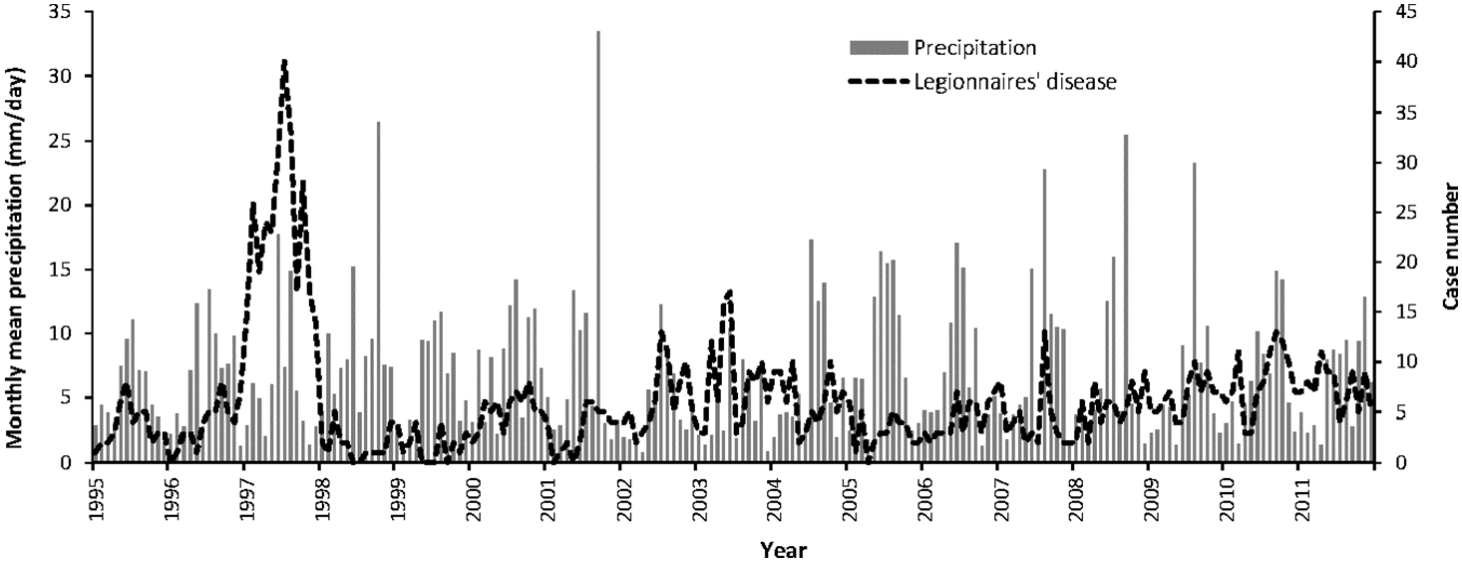
The monthly variability of Legionnaires’ disease occurrence and mean precipitation (mm/day) in Taiwan.

According to the monthly variability of LD occurrence and mean precipitation (mm/day) (Fig. 2), the peak of reported LD cases usually accompanied high precipitation levels. Thus, we speculate there may be a relationship between LD occurrence and precipitation.

**Figure 3:**
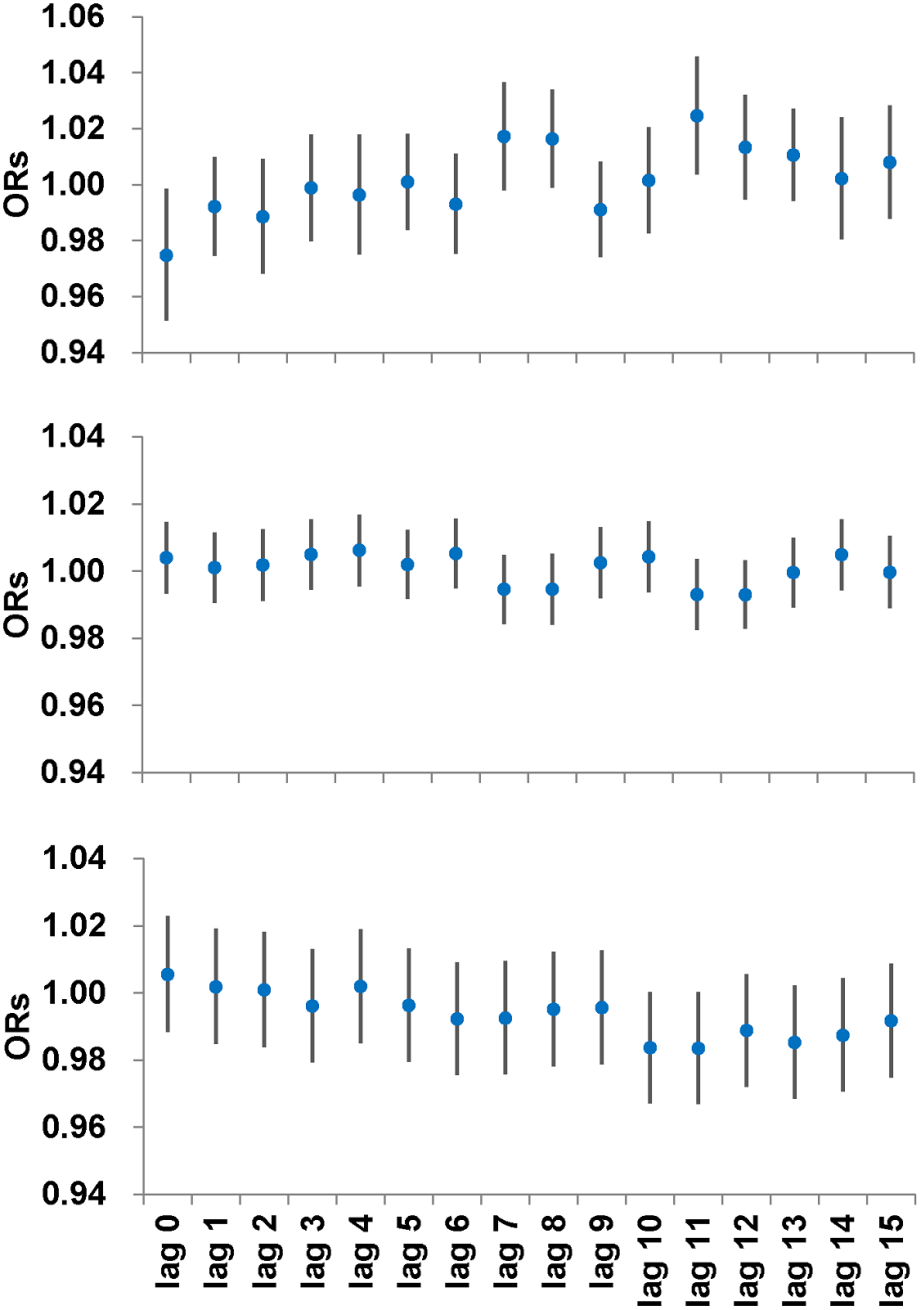
The adjusted ORs for occurrence of cases, based on an increase in 5 mm precipitation, each relative humidity and temperature at daily lag 0 to 15.

In this study, conditional logistic regressions were developed to evaluate the effects of daily temperature, relative humidity and precipitation on LD occurrence. The results indicated that temperature and precipitation, but not RH, may significantly affect LD occurrence (Figure 3). LD occurrence was negatively correlated with the mean temperature at the 10- and 11-day lag (*p*-value<0.05, Figure 3). With each degree Celsius rise in mean temperature, the incidence risk of reported LD cases was decreased by 3.2% (OR = 0.968, 95% CIs = 0.946–0.994) for lag day 10 and 2.5% (OR = 0.975, 95% CIs = 0.953–0.996) for lag day 11. In terms of daily temperature precipitation, an increase of 5 mm at lag 11 was associated with a 2.5% (95% CIs = 1.003–1.047, *p*-value = 0.02) increase in LD occurrence. Moreover, with each 10 mm increase in precipitation, the OR increase was increased by 5.0% (95% CIs = 0.6–9.6%) (data not shown).

We further separated the cases based on gender, age (less than 50 years/≧50 years), and season (cool: Oct–Dec and Jan–Feb and warm: Mar–Sep) to assess the effects of weather factors on LD occurrence. Positive and significant associations were only shown in the high risk group, i.e. male, ≧50 years, and cool season (Table 3). A 5-mm rise of precipitation at lag day-11 significantly enhanced the risk of LD occurrence by 2.3–2.9%. However, in the case of temperature, no significant association was found, regardless of the classification (*P*>0.05) (data not shown).

**Table 3:** Odds ratios (ORs) of a 5-mm increase of precipitation on the occurrence of Legionnaires’ disease at 10–13 days lag.

Variable	Lag 10		Lag 11		Lag 12		Lag 13	
	OR (95% CI)	*P*-value	OR (95% CI)	*P*-value	OR (95% CI)	*P*-value	OR (95% CI)	*P*-value
**Season** a								
** Warm**	1.007 (0.985, 1.030)	0.524	**1.029 (1.002, 1.056)**	**0.033** *	**1.030 (1.000, 1.061)**	**0.046** *	1.011 (0.989, 1.033)	0.343
** Cold**	0.988 (0.951, 1.027)	0.535	1.017 (0.982, 1.052)	0.342	1.002 (0.978, 1.027)	0.843	1.011 (0.986, 1.037)	0.382
**Gender**								
** Male**	1.003 (0.983, 1.024)	0.761	**1.024 (1.001, 1.047)**	**0.043** *	**1.023 (1.001, 1.047)**	**0.036** *	1.010 (0.992, 1.027)	0.278
** Female**	0.986 (0.938, 1.036)	0.565	1.024 (0.976, 1.074)	0.334	0.978 (0.931, 1.028)	0.385	1.014 (0.966, 1.063)	0.579
**Age**								
** <50**	1.004 (0.944, 1.068)	0.890	1.040 (0.976, 1.108)	0.225	0.999 (0.944, 1.058)	0.984	1.031 (0.978, 1.087)	0.251
** ≧50**	1.001 (0.982, 1.021)	0.903	**1.023 (1.001, 1.045)**	**0.040** *	1.015 (0.996, 1.035)	0.127	1.008 (0.991, 1.026)	0.342

a Warm season: March–August; cool season: September–December and January–February.

*Statistically significant, *P*<0.05.

**Figure 4:**
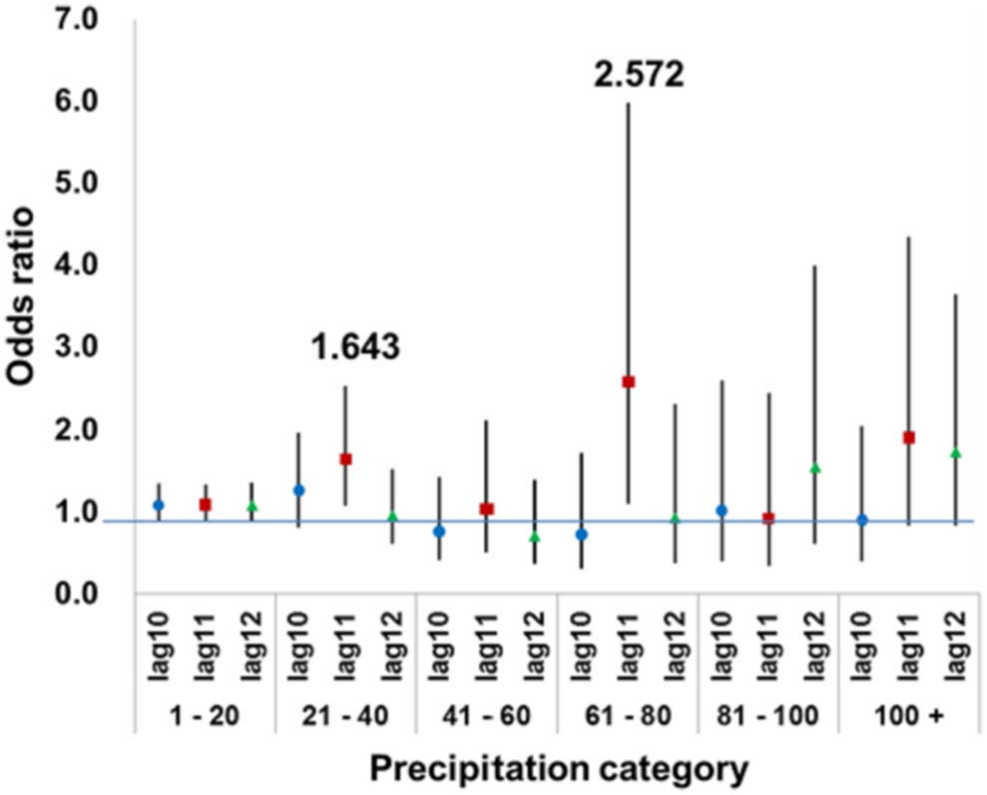
The estimated ORs for the precipitation categories at daily lag 10 to 12. Models were adjusted for temperature and relative humidity.

Our analysis indicated that higher precipitation 11 days prior to onset would increase the risk of LD occurrence (Figure 3). To clarify the effects of precipitation quantity on LD occurrence, we attemped to categorize the precipitation for each 20 mm increases up to 100 mm, and created conditional logistic regressions to assess the associations between reported LD cases and precipitation levels at 10–12 days prior to onset of these cases. Adjusted odds ratios for each precipitation category, which were relative to the baseline control precipitation category (0 mm), are presented in Figure 4. It can be seen that Precipitation 11 days before onset at light (21–40 mm) and heavy levels (61–80 mm) significantly increased the risk of LD occurrence by 1.643 (95% CIs = 1.074–2.513, *p*-value = 0.021) and 2.572 (95% CIs = 1.106–5.978, *p*-value = 0.028). These results suggest that reported LD cases increased with specific rainfall levels rather than with continuous increases in precipitation and indicate that risk levels of precipitation for LD occurrence occur at an 11-day lag.

## Discussion

The present study found that every 5-mm and 10-mm increases in precipitation at lag 11 corresponded to an increase in LD occurrence by 2.5 and 5.0%, respectively (Figure 3). This positive correlation between precipitation and LD is consistent with previous studies [12], [14], [18]. Moreover, the 11-day lag effect of precipitation agrees with Fisman’s study, which identified an acute positive effect of precipitation 6–10 days before legionellosis onset [12]. In this study, such an effect of precipitation at lag day-11 was repeatedly observed in men and elderly people who were susceptible to legionellae infection (Table 3). By considering the incubation period of 2–10 days for LD, patients are very likely to become infected on rainy days. The positive relationship between LD and precipitation was explained previously where it was shown that rainfall increases organic sediments and contamination with other microbes [19]–[20]. This assists legionellae replication and leads to an increase in the legionellae burden in tap water [12], [14]. However, if this pathway is the most important one, the lag period should be longer than those found both in our study and in previous studies because legionellae growth and transmission take time. Moreover, Rivera et al. found an inverse relationship between monthly precipitation and legionellae isolation from water collected from the faucets of healthcare facilities [21]. Therefore, it may be possible that patients inhale aerosols containing legionellae and become infected during rainy days. Since legionellae are frequently detected in puddles of rainwater on asphalt roads [22], we speculate that legionellae in soil [23]–[24], shade tree potting soil [25], or other habitats may be flushed out by rainfall, and then contaminate rainwater puddles on asphalt roads. The legionellae in puddles on roads are then aerosolized by moving vehicles, increasing the exposure risk to legionellae for people on the street.

To obtain a more detailed understanding of the impacts of precipitation on LD occurrence, in this study, daily precipitation (1 mm to >100 mm) was divided into five categories in order to compare ORs with the baseline control precipitation category (0 mm) (Figure 4). The positively significant association with reported LD cases only appeared in precipitation at 21–40 and 61–80 mm rather than with a continuous increase in precipitation. At these precipitation levels, rainwater puddles will form on roads, and people still go out, which may increase the opportunity of exposure to rainwater containing legionellae. As for heavier rainfall, during which legionellae in aquatic systems can be diluted, habitats of legionellae may be destroyed, and the frequency of going outside could be decreased, which might explain the non-significance of precipitation at >80 mm. Although our results are very preliminary, and there is a lack of evidence to support these speculations, the findings at least warn physicians to consider LD when choosing diagnostic tests and medication for patients showing signs and symptoms of community-acquired pneumonia 11 days after precipitation of 21–80 mm occurs.

It was also observed in the present study that temperature is negatively associated with reported LD cases (Figure 3), which was the opposite the finding of previous studies [12]–[13], [15]. However, this does not mean low temperature favors LD occurrence because more cases (48%) in Taiwan are reported during the warm summer and early autumn seasons (June to October) (data not shown), thus indicating that warm temperatures are still advantageous to LD occurrence. The negative correlation between temperature and LD occurrence might be explained by the significantly negative association of temperature with precipitation (*r* = –0.250 for June to October and –0.033 for November to May, *p*-value<0.05), i.e. precipitation increases reported LD cases but decreases temperature, which could be evident by the same 11-day lag effect for temperature and precipitation (Figure 3). Most importantly, when the data were separated into warm and cool seasons and reanalyzed, the impact of temperature was no longer significant in either season under consideration; however, during the warm season, higher precipitation at lag day-11 was still significantly correlated with greater LD occurrence (Table 3). These results seem to imply precipitation is a more critical factor for LD occurrence than temperature during the warm season in Taiwan.

RH is also a risk factor of LD occurrence and has been reported previously [13], [15], [17] although such a relationship was not significant in the study of Conza et al. [16]. Its significance was not found in this study (Figure 3), which may be because of the RH characteristic in Taiwan (Table 2). In Taiwan, the 5^th^ and 75^th^ percentiles for RH were at 63% and 82%, respectively (Table 2), indicating that RH levels on most days are close to the RH supporting the greatest legionellae survival (65 and 80%) [26]–[27]. Overall, the present study further suggests precipitation may be the major risk factor of LD occurrence in warm, humid regions.

A time-stratified case-crossover design was used in this study to assess the effects of weather factors on LD occurrence, which allowed joint analysis of many cities and eliminated the estimation of each city for executing meta-analysis. This design would be limited to presenting the entire long-term pattern between weather variation and LD occurrence in a time-series analysis. However, the simulation study indicated that a time-stratified case-crossover design may have greater statistical power than time-series analysis in the case of longitudinal studies [28].

## Conclusions

In the present study, the influences of temperature, RH, and precipitation on reported sporadic LD cases were evaluated, and it was found that in warm, humid regions, precipitation may be the most important weather factor enhancing LD occurrence in elderly male individuals during the warm season, especially at an 11-day lag. However, such a relationship is not completely linear. Precipitation at 21–40 and 61–80 mm may likely to impose greater risk of LD occurrence. These findings warn that 11 days after precipitation of 21–80 mm, LD should be carefully considered when diagnosing and medicating patients showing signs and symptoms of community-acquired pneumonia, especially in the case of elderly men in warm, humid regions.

## References

[1] Den Boer JW, Yzerman EP, Schellekens J, Boshuizen HC, Van Steenbergen JE, et al (2002) A large outbreak of Legionnaires’ disease at a flower show, the Netherlands, 1999. Emerg Infect Dis 8:37–43. 11749746 10.3201/eid0801.010176 PMC2730281

[2] Heath CH, Grove DI, Looke DF (1996) Delay in appropriate therapy of *Legionella pneumonia* associated with increased mortality. Eur J Clin Microbiol Infect Dis 15:286–290. 8781878 10.1007/BF01695659

[3] Joseph CA, Ricketts KD (2010) on behalf of the European Working Group for *Legionella* Infections. Legionnaires disease in Europe 2007–2008. Euro Surveill 15:19493. 29183473 10.2807/esm.09.10.00480-en

[4] Lettinga KD, Verbon A, Weverling GJ, Schellekens JFP, Den Boer JW, et al (2002) Legionnaires’ disease at a Dutch flower show: prognostic factors and impact of therapy. Emerg Infect Dis 8:1448–1454. 12498662 10.3201/eid0812.020035 PMC2738521

[5] Dominguez A, Alvarez J, Sabria M, Torner N, Oviedo M, et al (2009) Factors influencing the case-fatality rate of Legionnaires’ disease. Int J Tuberc Lung Dis 13:407–412. 19275805

[6] Helms CM, Viner JP, Weisenburger DD, Chiu LC, Renner ED, et al (1984) Sporadic Legionnaires’ disease: clinical observations on 87 nosocomial and community-acquired cases. Am J Med Sci 288:2–12. 6465187 10.1097/00000441-198407000-00001

[7] Marston BJ, Lipman HB, Breiman RF (1994) Surveillance for Legionnaires’ disease. Risk factors for morbidity and mortality. Arch Intern Med 154:2417–2422. 7979837

[8] Jones TF, Benson RF, Brown EW, Rowland JR, Crosier SC, et al (2003) Epidemiologic investigation of a restaurant-associated outbreak of Pontiac fever. Clin Infect Dis 37:1292–1297. 14583861 10.1086/379017

[9] Ferre MR, Arias C, Oliva JM, Pedrol A, Garcia M, et al (2009) A community outbreak of Legionnaires’ disease associated with a cooling tower in Vic and Gurb, Catalonia (Spain) in 2005. Eur J Clin Microbiol Infect Dis 28:153–159. 18752009 10.1007/s10096-008-0603-6

[10] Krojgaard LH, Krogfelt KA, Albrechtsen HJ, Uldum SA (2011) Cluster of Legionnaires’ disease in a newly built block of flats, Denmark, December 2008 - January 2009. Euro Surveill 16:19759. 21223834

[11] Pravinkumar SJ, Edwards G, Lindsay D, Redmond S, Stirling J, et al (2010) A cluster of Legionnaires’ disease caused by *Legionella longbeachae* linked to potting compost in Scotland, 2008–2009. Euro Surveill 15:19496. 20197024 10.2807/ese.15.08.19496-en

[12] Fisman DN, Lim S, Wellenius GA, Johnson C, Britz P, et al (2005) It’s not the heat, it’s the humidity: wet weather increases legionellosis risk in the greater Philadelphia metropolitan area. J Infect Dis 192:2066–2073. 16288369 10.1086/498248

[13] Karagiannis I, Brandsema P, VAN DER Sande M (2009) Warm, wet weather associated with increased Legionnaires’ disease incidence in The Netherlands. Epidemiol Infect 137:181–187. 18631425 10.1017/S095026880800099X

[14] Hicks LA, Rose CE, Fields BS, Drees ML, Engel JP, et al (2007) Increased rainfall is associated with increased risk for legionellosis. Epidemiol Infect 135:811–817. 17121693 10.1017/S0950268806007552 PMC2870637

[15] Ng V, Tang P, Jamieson F, Drews SJ, Brown S, et al (2008) Going with the flow: legionellosis risk in Toronto, Canada is strongly associated with local watershed hydrology. Ecohealth 5:482–490. 19370300 10.1007/s10393-009-0218-0

[16] Conza L, Casati S, Limoni C, Gaia V (2013) Meteorological factors and risk of community-acquired Legionnaires’ disease in Switzerland: an epidemiological study. BMJ Open 3:e002428. 10.1136/bmjopen-2012-002428 PMC3612760 23468470

[17] Ricketts KD, Charlett A, Gelb D, Lane C, Lee JV, et al (2009) Weather patterns and Legionnaires’ disease: a meteorological study. Epidemiol Infect 137:1003–1012. 19017428 10.1017/S095026880800157X

[18] Garcia-Vidal C, Labori M, Viasus D, Simonetti A, Garcia-Somoza D, Dorca J, et al (2013) Rainfall is a risk factor for sporadic cases of *Legionella pneumophila* pneumonia. PLoS One 8:e61036. 23613778 10.1371/journal.pone.0061036 PMC3628787

[19] Richardson HY, Nichols G, Lane C, Lane C, Lake IR, et al 2009. Microbiological surveillance of private water supplies in England: the impact of environmental and climate factors on water quality. Water Res 43:2159–2168. 19303126 10.1016/j.watres.2009.02.035

[20] Atherholt TB, LeChevallier MW, Norton WD, Rosen JS (1998) Effect of rainfall on Giardia and crypto. J Am Water Works Ass 90:66–80.

[21] Rivera JM, Granizo JJ, Aguilar L, Gimenez MJ, Aguiar JM, et al (2009) Is there a relationship between monthly rainfall and the isolation of *Legionella* in potable water systems in Spanish healthcare facilities? Infect Control Hosp Epidemiol 30:306–308. 19215199 10.1086/595981

[22] Sakamoto R, Ohno A, Nakahara T, Satomura K, Iwanaga S, et al (2009) *Legionella pneumophila* in rainwater on roads. Emerg Infect Dis 15:1295–1297. 19751596 10.3201/eid1508.090317 PMC2815982

[23] Travis TC, Brown EW, Peruski LF, Tatiana C, Siludjai D, et al (2012) Survey of *Legionella* species found in thai soil. Int J Microbiol 2012:218791. 22287969 10.1155/2012/218791 PMC3263619

[24] Hong PY, Yannarell AC, Dai Q Ekizoglu M, Mackie RI (2013) Monitoring the perturbation of soil and groundwater microbial communities due to pig production activities. Appl Environ Microbiol 79:2620–2629. 23396341 10.1128/AEM.03760-12 PMC3623201

[25] Casati S, Gioria-Martinoni A, Gaia V (2009) Commercial potting soils as an alternative infection source of *Legionella pneumophila* and other *Legionella* species in Switzerland. Clin Microbiol Infec 15:571–575. 19392903 10.1111/j.1469-0691.2009.02742.x

[26] Hambleton P, Broster MG, Dennis PJ, Henstridge R, itzgeorge R, et al (1983) Survival of virulent *Legionella pneumophila* in aerosols. J Hyg (Lond) 90:451–460. 6863914 10.1017/s0022172400029090 PMC2134264

[27] Berendt RF (1980) Survival of *Legionella pneumophila* in aerosols: effect of relative humidity. J Infect Dis 141:689. 7373091 10.1093/infdis/141.5.689

[28] Figueiras A, Carracedo-Martinez E, Saez M, Taracido M (2005) Analysis of case-crossover designs using longitudinal approaches: a simulation study. Epidemiology. 16 239–246:1. 10.1097/01.ede.0000152915.58564.d3 15703540

